# Categorization of Upper Gastrointestinal Symptoms Is Useful in Predicting Background Factors and Studying Effects and Usages of Digestive Drugs

**DOI:** 10.1371/journal.pone.0088277

**Published:** 2014-02-05

**Authors:** Nobutake Yamamichi, Takeshi Shimamoto, Yoshiki Sakaguchi, Yu Takahashi, Shinya Kodashima, Chiemi Nakayama, Chihiro Minatsuki, Satoshi Ono, Satoshi Mochizuki, Rie Matsuda, Itsuko Asada-Hirayama, Keiko Niimi, Mitsuhiro Fujishiro, Yosuke Tsuji, Chihiro Takeuchi, Hikaru Kakimoto, Osamu Goto, Toru Mitsushima, Kazuhiko Koike

**Affiliations:** 1 Department of Gastroenterology, Graduate School of Medicine, The University of Tokyo, Tokyo, Japan; 2 Department of Gastroenterology, Kameda Medical Center Makuhari, Chiba, Japan; University Hospital Llandough, United Kingdom

## Abstract

**Background:**

There have been very few reports assessing the relationship between various upper gastrointestinal (GI) symptoms or evaluating each individual upper GI symptom separately.

**Methods:**

Based on the answers to Frequency Scale for the Symptoms of GERD from a large-scale population of healthy adults in Japan, a hierarchical cluster analysis was performed to categorize the typical 12 upper GI symptoms. The associations between the 12 symptoms and 13 background factors were systematically analyzed among the 18,097 digestive drug-free subjects, 364 proton-pump inhibitor (PPI) users, and 528 histamine H_2_-receptor antagonist (H_2_RA) users.

**Results:**

The derived relationship between the 12 upper GI symptoms suggests the five symptom categories: heartburn (2), dyspepsia (4), acid regurgitation (3), pharyngo-upper esophageal discomfort (2), and fullness while eating (1). Among the digestive drug-free subjects, inadequate sleep, weight gain in adulthood, NSAID use, meals immediately prior to sleep, and frequent skipping of breakfast showed significant positive association with most upper GI symptoms. Compared to the digestive drug-free subjects, significantly associated factors for PPI and H_2_RA users are respectively different in “4 of 5” and “5 of 5” symptoms in heartburn and acid regurgitation categories, “1 of 2” and “1 of 2” symptoms in pharyngo-upper esophageal discomfort category, and “0 of 5” and “3 of 5” symptoms in dyspepsia and fullness while eating categories. These differences between digestive drug-free subjects and gastric acid suppressant users seem to correlate with our experiences in clinical situations: heartburn and acid regurgitation category symptoms are effectively controlled with PPI and H_2_RA whereas other category symptoms are not.

**Conclusions:**

The 12 upper GI symptoms can be classified into five categories, which are statistically associated with various background factors. The differences of associated factors between digestive drug-free subjects and digestive drug users may be useful in studying the drug effects upon diverse upper GI symptoms.

## Introduction

The term “upper gastrointestinal (GI) symptoms” is commonly used to describe multiple complaints including heartburn, regurgitation, postprandial fullness, early satiety, epigastric pain, belching, nocturnal pain, fasting pain, nausea and vomiting, abdominal distention, and so on [1]. There have been many previous reports concerning upper GI symptoms which focused on the three symptom categories separately: gastroesophageal reflux symptoms [2], [3], [4], [5], [6], [7], [8], dyspeptic symptoms [7],[9],[10],[11],[12],[13], and peptic ulcer related symptoms [7], [14], [15], [16]. However, there have been very few reports assessing the relationship between various upper GI symptoms. In the present study, we therefore tried to statistically categorize the typical upper GI symptoms using a hierarchical cluster analysis.

Of the many upper GI symptoms, gastroesophageal reflux disease (GERD) symptoms are thought to be the most common [17], [18]; the prevalence of reflux esophagitis (RE) and non-erosive reflux disease (NERD) are respectively 15.5% and 27.1% in Sweden [8], 6.8% and 15.9% in Japan [5]. Although GERD patients present a diverse range of symptoms including extraesophageal symptoms [3], [6], [19], it is broadly accepted that the most typical symptoms of GERD are heartburn and regurgitation [4]. Dyspeptic symptoms are also thought to be very common [17], [18]; the prevalence of functional dyspepsia was reported to be 14.7% in Norway [20], [21] and 11.5% in England [22]. Though an accurate evaluation concerning the prevalence of dyspepsia is difficult, it is thought to be in the range of 10–40% [9]. We previously evaluated the associations of FSSG (Frequency Scale for the Symptoms of GERD) scores [19] with multiple lifestyle related factors using the data from a large-scale cohort of 19,864 healthy adults [3]. Although the FSSG questionnaire was originally developed for evaluating symptoms of GERD patients [19], the 12 questions of FSSG target not only “acid reflux-related symptoms” but also “dyspeptic (dysmotility) symptoms” [23]. Thus, it has been used for not only evaluation of GERD symptoms [3], [24], [25] but also for evaluating functional dyspepsia (FD) [26]. Consequently, we used the 12 symptoms included in the FSSG as the typical upper GI symptoms in the present study.

In our recent report [3], we found that the total FSSG score is significantly associated with many lifestyle related factors such as inadequate sleep, increased body weight in adulthood, meals immediately prior to sleep, midnight snacks, body mass index (BMI), frequent skipping of breakfast, lack of habitual physical exercise, quick eating, etc. However, we had not performed the thorough analyses evaluating association between individual upper GI symptoms and putative background factors separately (systemic analyses). Many questionnaires assessing diverse upper GI symptoms have been proposed [3], [19], [27], [28], [29], but detailed systemic evaluation of individual upper GI symptoms had not been executed. In this study, we therefore analyzed the individual 12 upper GI symptoms separately, together with putative background factors identified in our previous reports [3], [5], [30].

Based on the results from our recent analyses [3], [5], [31], we have decided to analyze the following 13 background factors: age, gender, BMI, serum *Helicobacter pylori (HP)* IgG, ratio of serum pepsinogen I/II reflecting atrophy of gastric mucosa, use of NSAIDs, inadequate sleep, weight gain in adulthood, intake of meals immediately prior to sleep, frequent skipping of breakfast, lack of habitual exercise, habitual alcohol drinking, and habitual smoking. In our present study, we tried to evaluate not only the persons free from digestive-drug use, but also the proton pump inhibitor (PPI) users and histamine H_2_-receptor antagonist (H_2_RA) users. These two drugs are the most popular gastric acid suppressants used for upper GI disorder including GERD [3], [32], [33], peptic ulcer disease [14], [34], [35], and dyspepsia [10], [36], [37]. Therefore, we hypothesized that a comparison of the background factors of PPI and H_2_RA users with those of digestive drug-free subjects might be useful in predicting the efficacy of controlling intragastric pH upon various upper GI symptoms.

## Materials and Methods

### Study Subjects

All the subjects who received medical checkup at Kameda Medical Center Makuhari (Chiba-shi, Chiba, Japan) during the year 2010 were asked to participate in our study. All subjects were physically self-reliant healthy outpatients, who voluntarily applied for a complete physical examination at our institute. A total of 20,773 subjects (50.2±9.5 years of age) assented and were enrolled in our study. In cases where health checkup was performed twice in 2010, the results from the former checkup were used. Cases less than 20 years of age, with a medical history of gastrectomy, and with insufficient data for analysis were excluded from this study. This study was approved by the ethics committee of the University of Tokyo, and written informed consent was obtained from all the study participants according to the Declaration of Helsinki.

### Frequency Scale for the 12 Upper GI Symptoms and Questionnaire about Lifestyles

**Figure 1:**
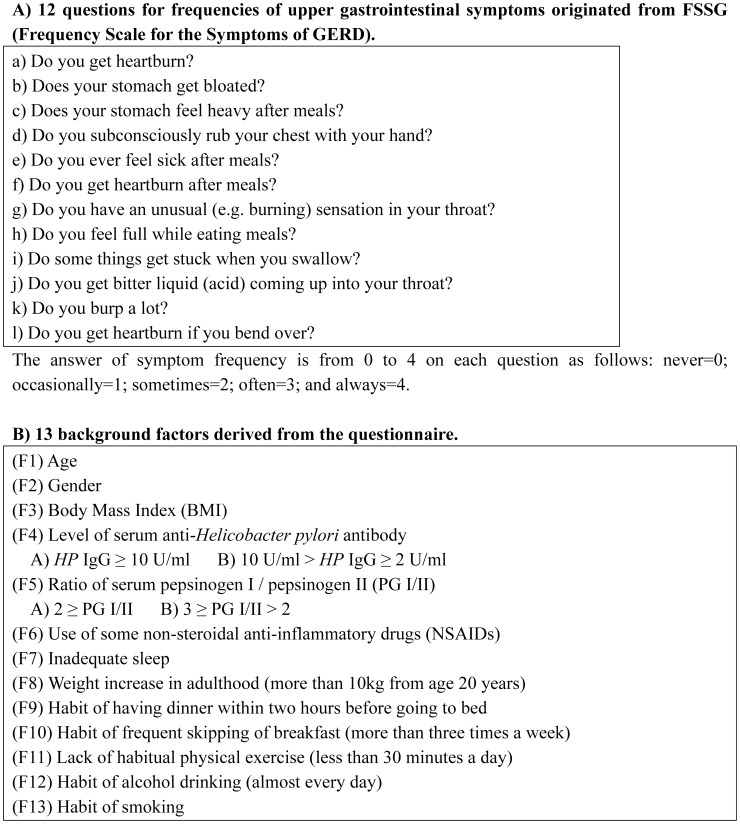
The 12 questions for frequencies of various upper gastrointestinal symptoms (A) and 13 background factors derived from the questionnaire (B).

To assay various upper GI symptoms, we analyzed the 12 symptom scores included in the Frequency Scale for the Symptoms of GERD (FSSG), which is a validated and widely used questionnaire covering various symptoms related to the upper gastrointestinal tract [3], [19], [23]. The frequency of each of these 12 upper GI symptoms (Figure 1A) was measured on the following scale: never = 0; occasionally = 1; sometimes = 2; often = 3; and always = 4. As the response variables for the statistical analyses, we used the 12 symptom scores derived from the study subjects.

For the explanatory variables, we adopted 13 factors based on our past research [3], [5], [30], [31] (Figure 1B). We selected age (F1), gender (F2), and BMI (F3) as the three basic factors, and serum *HP* IgG (F4) and ratio of serum pepsinogen I/II (F5) as gastric mucosa-related factors. In addition, we selected drinking (F12), smoking (F13), and the six following yes-no questionnaire filled in by all participants (Figure 1B); (F6) Do you take any non-steroidal anti-inflammatory drugs (NSAIDs)?; (F7) Do you feel you do not have adequate sleep?; (F8) Has your body weight markedly increased in adulthood (more than 10 kg from the age of 20 years)?; (F9) Do you habitually have a midnight snack (more than three times a week)?; (F10) Do you frequently skip breakfast (more than three times a week)?; and (F11) Is your time of exercise less than 30 minutes a day?

### Statistical Methods

A hierarchical cluster analysis (Ward’s method with Euclidean distances) was performed in order to group the 12 upper GI symptoms based on the questionnaire answers from the digestive drug-free subjects. The results of cluster analyses were computed into a cluster dendrogram, which became the basis of our systemic categorization of multiple upper GI symptoms.

Correlation analyses were exhaustively performed, using the 12 upper GI symptoms as response variables and the above-mentioned 13 background factors as explanatory variables (systemic analyses). Digestive drug-free subjects, PPI users, and H_2_RA users were analyzed separately. For univariate systemic analyses, Student’s t-test or Pearson’s correlation coefficient were applied. For multivariate systemic analyses, the multiple linear regression model was applied to relevant background factors for each of the 12 response variables. The effect sizes (f^2^) and power of all the variables were also calculated. In both univariate and multivariate systemic analyses, two-sided *p* values of less than 0.005 (for digestive drug-free subjects) or 0.05 (for PPI users and H_2_RA users) were considered statistically significant.

To assess the association between various background factors of digestive drug-free subjects and gastric acid suppressant (PPI or H_2_RA) users, analysis of covariance (ANCOVA) was additionally performed, in which *p* values of less than 0.01 were considered statistically significant. All statistical analyses were performed using SAS version 8.2 (SAS Institute Inc., Cary, NC, USA) or JMP version 8.0 (SAS Institute Inc.) software.

## Results

### Characteristics of the Study Subjects and 12 Upper Gastrointestinal Symptoms

**Figure 2:**
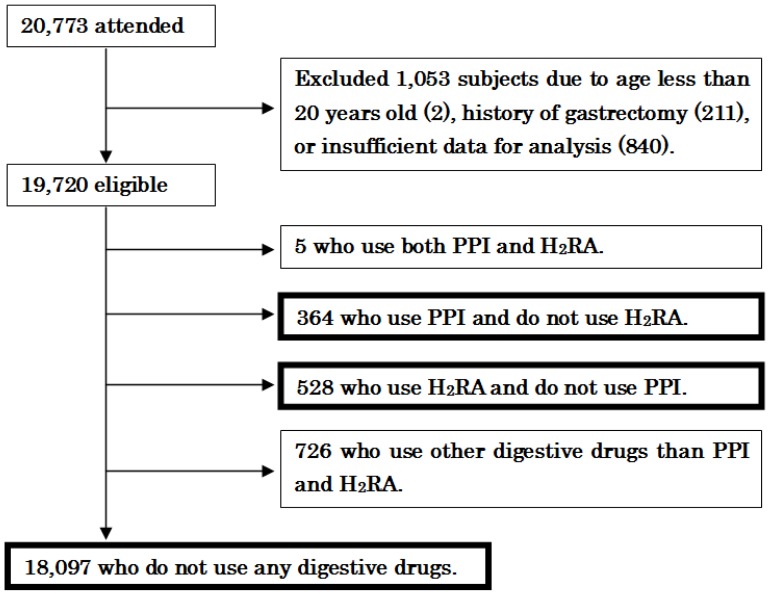
Study recruitment flowchart.

Of the 20,773 subjects who were originally enrolled in this study, we excluded 1,053 subjects due to an age of less than 20 years old (2), a history of gastrectomy (211), or insufficient data for analysis (840). As shown in Figure 2, the eligible 19,720 subjects comprised of 5 subjects using both PPI and H_2_RA, 364 PPI users who do not use H_2_RA (236 men and 128 women with a mean age of 55.6±9.9 years), 528 H_2_RA users who do not use PPI (323 men and 205 women with a mean age of 52.8±9.7 years), 726 subjects using digestive drug other than PPI and H_2_RA (393 men and 333 women with a mean age of 52.4±9.4 years), and 18,097 digestive drug-free subjects who do not use any digestive drugs (10,406 men and 7,691 women with a mean age of 49.8±9.3 years).

Scores of the 12 upper GI symptoms among the 18,097 digestive drug-free subjects, 364 PPI users, and 528 H_2_RA users are shown in Table 1. For all the 12 upper GI symptoms, scores of PPI users and H_2_RA users are significantly higher than those of digestive drug-free subjects.

**Table 1:** Scores of the typical 12 upper GI symptoms among the 18,097 digestive drug-free subjects, 364 PPI users, and 528 H_2_RA users.

12 upper gastrointestinal symptoms	18,097 digestive drug-free subjects	364 PPI users	528 H_2_RA users
a) Getting heartburn	0.51±0.78	1.29±1.14	1.13±1.08
b) Stomach getting bloated	0.79±0.96	1.10±1.10	1.14±1.10
c) Stomach feeling heavy	0.57±0.81	1.08±1.13	1.17±1.07
d) Rubbing the chest with hands	0.18±0.51	0.44±0.82	0.43±0.81
e) Feeling sick after meals	0.20±0.50	0.38±0.70	0.43±0.75
f) Getting heartburn after meals	0.38±0.66	0.89±1.04	0.80±0.97
g) Unusual sensation in the throat	0.33±0.75	0.66±1.04	0.47±0.91
h) Feeling full while eating the meals	0.31±0.64	0.46±0.78	0.47±0.82
i) Some thing getting stuck in swallowing	0.19±0.52	0.38±0.76	0.26±0.57
j) Bitter liquid coming up to the throat	0.37±0.65	0.91±1.04	0.73±0.87
k) Burping a lot	0.52±0.87	0.94±1.11	0.88±1.09
l) Getting heartburn while bending over	0.13±0.44	0.48±0.94	0.32±0.70
Total of 12 symptom scores	4.46±4.95	9.00±7.35	8.23±6.57

Scores of the 12 upper GI symptoms included in the FSSG range from 0 to 4 respectively. Consequently, total symptom scores range from 0 to 48.

### Proposal of Five Categories for the 12 Upper GI Symptoms

**Figure 3:**
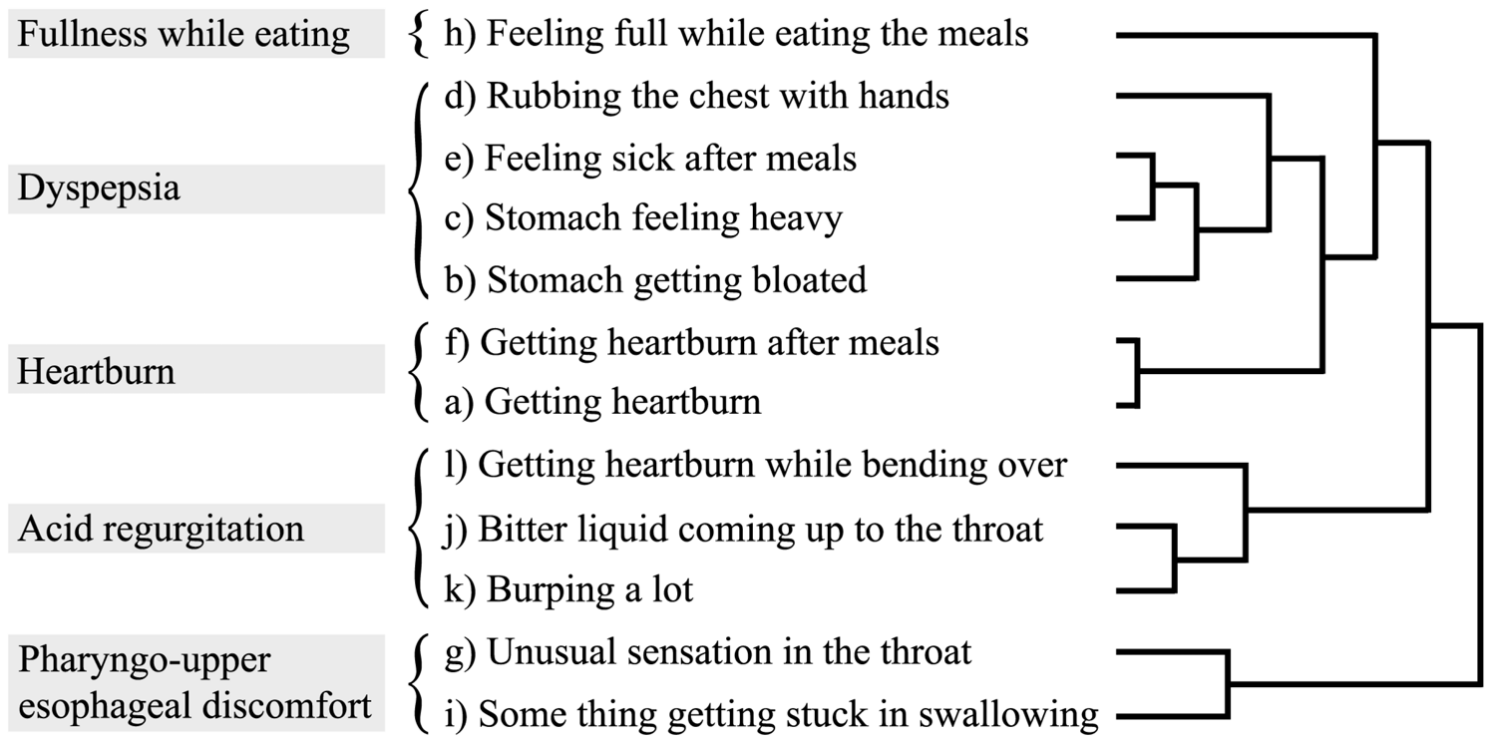
A dendrogram of the 12 upper GI symptoms based on the hierarchical cluster analysis (Ward’s method) of the 18,097 digestive drug-free subjects. Our proposed five categorization of 12 upper GI symptoms is also denoted.

To analyze the interrelation among the 12 upper GI symptoms, a hierarchical cluster analysis was performed based on the data from 18,097 digestive drug-free subjects. The result is visualized as a dendrogram (Figure 3), which denotes “distances” among the 12 symptoms. Although the 12 upper GI symptoms included in the FSSG [19] have been originally classified into acid reflux-related (a, d, f, g, i, j, and l) and dyspeptic (b, c, e, h, and k) symptoms [23], our results do not completely conform to this categorization. For example, “j) bitter liquid coming up to the throat” (belonging to acid reflux-related symptoms) and “k) burping a lot” (belonging to dyspeptic symptoms) are relatively close. For another example, “g) unusual sensation in the throat” and “i) some thing getting stuck in swallowing”, both originally considered to be acid reflux-related symptoms, are not closely related to other acid reflux-related symptoms.

Based on the cluster dendrogram, we propose that the 12 upper GI symptoms can be classified into five categories (Figure 3): heartburn (f and a), dyspepsia (d, e, c, and b), acid regurgitation (l, j, and k), pharyngo-upper esophageal discomfort (g and i), and fullness while eating (h). Whereas heartburn and acid regurgitation are considered as two of the most typical GERD symptoms [6], our results suggest that these two should be treated separately (Figure 3). Dyspepsia, one of the most common symptoms of functional gastrointestinal disorders [9], [18], [20], [38], includes three typical dyspeptic symptoms (b, c, and e) [23], [39] and one unexpected symptom (d). Pharyngo-upper esophageal discomfort includes two close symptoms (i and g), which may be considered as extraesophageal GERD symptoms [19], [40]. Contrary to prior belief, fullness while eating (h) is not closely related to any of the other dyspeptic symptoms (b, c, and e); it is in fact the most isolated symptom among the 12 upper GI symptoms (Figure 3).

### Background Factors for the Individual 12 Upper GI Symptoms among the 18,097 Digestive Drug-free Subjects, 364 PPI Users, and 528 H_2_RA Users

Distribution of the 12 upper GI symptom scores and 13 background factors are shown as histograms (Figure S1 and S2) based on the data of 18,097 digestive drug-free subjects. Of the 13 analyzed factors, use of NSAIDs (F6), lack of habitual exercise (F11), inadequate sleep (F7), frequent skipping of breakfast (F10), and meals immediately prior to sleep (F9) seem to be risk factors for all 12 upper GI symptoms. The other eight factors do not show unidirectional tendencies: both positive and negative associations are observed for the 12 upper GI symptoms (Figure S1 and S2).

Using the data from the 18,097 digestive drug-free subjects, univariate and multivariate analyses were further performed to evaluate associations between the 12 symptoms and 13 background factors exhaustively. The results of systematic univariate analyses (Table S1) show various associations between the 13 causative factors and the 12 upper GI symptoms. Among the 13 factors, inadequate sleep (F7) is apparently the strongest associated risk factor for most upper GI symptoms, which is consistent with our recent report [3]. The following systematic multivariate analyses (Table 2) also confirmed that inadequate sleep (F7) is the strongest risk factor for 11 of 12 upper GI symptoms. In addition, weight gain in adulthood (F8), use of NSAID (F6), meals immediately prior to sleep (F9), and frequent skipping of breakfast (F10) also showed significant positive associations with almost all 12 symptoms (Table 2). We designated these five as common risk factors for upper GI symptoms.

**Table 2:** Orders, directions, and standardized coefficients of association between the 12 upper GI symptoms and 13 background factors among the 18,097 digestive drug-free subjects.

Factors	F1	F2	F3	F4A	F4B	F5A	F5B	F6	F7	F8	F9	F10	F11	F12	F13
h) Feeling fullwhile eatingthe meals	4N	2P	1N		10P			7P	3P	8P		5P		9P	6P
	0.053	0.088	0.101		0.022			0.041	0.081	0.039		0.051		0.026	0.042
d) Rubbing thechest with hands		5P	7N					4P	1P	2P	3P	6P	8P		
		0.038	0.030					0.040	0.087	0.047	0.044	0.031	0.025		
e) Feeling sickafter meals	2N	3P	4N	9P				6P	1P	5P	7P	8P			
	0.094	0.084	0.051	0.035				0.047	0.106	0.050	0.043	0.038			
c) Stomachfeeling heavy	4N	2P	3N	9P	10P			7P	1P	5P	6P	8P			
	0.067	0.082	0.070	0.040	0.028			0.054	0.123	0.063	0.055	0.042			
b) Stomachgetting bloated	7N	2P	5N					4P	1P	3P	6P	8P	9P		
	0.043	0.114	0.061					0.065	0.130	0.069	0.054	0.032	0.022		
f) Gettingheartburnafter meals		8P		2P	7P	4N		5P	1P	3P	6P	9P		10P	
		0.035		0.083	0.037	0.053		0.045	0.103	0.060	0.045	0.032		0.030	
a) Gettingheartburn				2P	5P	4N	11N	6P	1P	3P	7P	8P		9P	10P
				0.079	0.046	0.055	0.032	0.046	0.104	0.064	0.041	0.035		0.034	0.033
l) Gettingheartburn whilebending over		3P						5P	1P	2P	4P				
		0.045						0.024	0.077	0.060	0.036				
j) Bitter liquidcoming upto the throat		3N		4P	11P	5N	9N	7P	1P	2P	6P	8P	10P		
		0.071		0.058	0.023	0.051	0.027	0.034	0.105	0.080	0.046	0.030	0.027		
k) Burping a lot	5N	2N	4N					6P	1P	3P	7P				
	0.036	0.077	0.038					0.031	0.081	0.044	0.028				
g) Unusualsensationin the throat	6P							3P	1P	2P	4P	8P	5P		7P
	0.027							0.044	0.095	0.045	0.033	0.024	0.031		0.026
i) Some thinggetting stuck inswallowing	2P	3P						6P	1P	4P		5P	7P		
	0.044	0.043						0.025	0.090	0.034		0.030	0.024		
Total 12 upperGI symptoms	9N	7P	6N	5P	10P	12N	14N	3P	1P	2P	4P	8P	11P	13P	
	0.034	0.052	0.052	0.056	0.033	0.030	0.025	0.071	0.164	0.090	0.065	0.050	0.033	0.024	

Background factors are (F1) age, (F2) female gender, (F3) BMI, (F4A) *HP* IgG ≥10 U/ml, (F4B) 10 U/ml>*HP* IgG ≥2 U/ml, (F5A) 2≥PG I/II, (F5B) 3≥PG I/II >2, (F6) use of NSAIDs, (F7) inadequate sleep, (F8) weight gain in adulthood, (F9) meals immediately prior to sleep, (F10) frequent skipping of breakfast, (F11) lack of habitual exercise, (F12) alcohol drinking, and (F13) smoking. (F4A) and (F4B) were compared with “2> *HP* IgG”, and (F5A) and (F5B) were compared with “PG I/II >3”. Orders of association among the 13 background factors are shown as the upper integers for the individual 12 symptoms, in which attached “P” and “N” denote positive and negative association respectively. Standardized coefficients are shown as the lower decimal fractions. The levels of significance in these multivariate analyses were set at <0.005.

Next, we performed multivariate analyses on the users of gastric acid suppressants, to evaluate the thorough associations between the 12 upper GI symptoms and 13 background factors (Table 3 for PPI users, Table 4 for H_2_RA users). The distributions of significant factors for PPI users and H_2_RA users are apparently different from those of digestive drug-free subjects (Table 2). In particular, serum *HP* IgG (F4), serum PG I/II ratio (F5), use of NSAID (F6), and weight gain in adulthood (F8) seldom showed significant association with upper GI symptoms among either PPI and H_2_RA users. Conversely, age (F1), sex (F2), and BMI (F3) among the digestive drug-free subjects and gastric acid suppressant users displayed similar associations with several upper GI symptoms (Table 2–4). It is intriguing that some significant factors for upper GI symptoms are markedly different between gastric acid suppressant users and digestive drug-free subjects, whereas others are similar.

**Table 3:** Orders, directions, and standardized coefficients of associations between the 12 upper GI symptoms and 13 background factors among the 364 PPI users.

Factors	*p* value	F1	F2	F3	F4A	F4B	F5A	F5B	F6	F7	F8	F9	F10	F11	F12	F13
h) Feeling fullwhile eatingthemeals	0.3738		1P	2N						3P						4P
			0.205	0.200						0.115						0.114
d) Rubbing thechest with hands	0.0856	5N	2P	4N						3P	1P	6P				
		0.124	0.154	0.148						0.149	0.223	0.117				
e) Feeling sickafter meals	0.1042	1N	3P	2N						4P						
		0.222	0.185	0.196						0.144						
c) Stomachfeelingheavy	0.0100	2N	1P	3N						4P			5P			
		0.147	0.218	0.140						0.131			0.112			
b) Stomachgettingbloated	0.0133	2N								1P						
		0.145								0.195						
f) Getting heartburnafter meals	<0.0001*		1P										2P			
			0.153										0.134			
a) Getting heartburn	<0.0001*	1N	2P		4N								3P			
		0.171	0.134		0.117								0.124			
l) Getting heartburnwhile bendingover	<0.0001*												1P			
													0.174			
j) Bitter liquidcoming up tothe throat	<0.0001*	2N	3P										1P			
		0.131	0.120										0.172			
k) Burping a lot	0.1720	1N										2P				
		0.128										0.121				
g) Unusualsensationin the throat	0.1482									1P						
										0.174						
i) Some thinggetting stuck inswallowing	0.0027*									2P		1P			3N	
										0.148		0.187			0.146	
Total 12 upperGI symptoms	<0.0001*	2N	1P							3P		5P	4P			
		0.177	0.185							0.172		0.138	0.142			

Background factors are (F1) age, (F2) female gender, (F3) BMI, (F4A) *HP* IgG ≥10 U/ml, (F4B) 10 U/ml>*HP* IgG ≥2 U/ml, (F5A) 2≥PG I/II, (F5B) 3≥PG I/II >2, (F6) use of NSAIDs, (F7) inadequate sleep, (F8) weight gain in adulthood, (F9) meals immediately prior to sleep, (F10) frequent skipping of breakfast, (F11) lack of habitual exercise, (F12) alcohol drinking, and (F13) smoking. (F4A) and (F4B) were compared with “2> *HP* IgG”, and (F5A) and (F5B) were compared with “PG I/II >3”. Orders of association among the 13 background factors are shown as the upper integers for the individual 12 symptoms, in which attached “P” and “N” denote positive and negative association respectively. Standardized coefficients are shown as the lower decimal fractions. The levels of significance in these multivariate analyses were set at <0.05. The differences of associated background factors between PPI users and digestive drug-free subjects were calculated; *p* scores below 0.05 were set for the level of significance.

**Table 4:** Orders, directions, and standardized coefficients of associations between the 12 upper GI symptoms and 13 background factors among the 528 H_2_RA users.

Factors	*p* value	F1	F2	F3	F4A	F4B	F5A	F5B	F6	F7	F8	F9	F10	F11	F12	F13
h) Feeling fullwhile eatingthe meals	0.1676		2P	1N			4N			5P		3P				
			0.132	0.170			0.100			0.092		0.104				
d) Rubbing thechest with hands	0.0056*									1P		2P				
										0.123		0.104				
e) Feeling sickafter meals	0.2000	3N	1P							2P						
		0.133	0.220							0.158						
c) Stomach feelingheavy	0.0078*		1P	2N						3P						
			0.174	0.174						0.170						
b) Stomach gettingbloated	0.0043*	4N	2P		3N					1P						
		0.100	0.138		0.113					0.244						
f) Getting heartburnafter meals	<0.0001*									1P						
										0.155						
a) Getting heartburn	<0.0001*									1P						
										0.190						
l) Getting heartburnwhile bending over	0.0024*					2N				1P						
						0.113				0.124						
j) Bitter liquidcoming upto the throat	0.0023*									1P			2P			
										0.175			0.100			
k) Burping a lot	0.0002*	2N								1P						
		0.112								0.161						
g) Unusual sensationin the throat	0.6368									1P						
										0.100						
i) Some thinggetting stuckin swallowing	0.0039*															
Total 12 upperGI symptoms	<0.0001*	3N	2P							1P						
		0.103	0.111							0.247						

Background factors are (F1) age, (F2) female gender, (F3) BMI, (F4A) *HP* IgG ≥10 U/ml, (F4B) 10 U/ml>*HP* IgG ≥2 U/ml, (F5A) 2≥PG I/II, (F5B) 3≥PG I/II >2, (F6) use of NSAIDs, (F7) inadequate sleep, (F8) weight gain in adulthood, (F9) dinner just before bedtime, (F10) frequent skipping of breakfast, (F11) lack of habitual exercise, (F12) alcohol drinking, and (F13) smoking. (F4A) and (F4B) were compared with “2> *HP* IgG”, and (F5A) and (F5B) were compared with “PG I/II >3”. Orders of association among the 13 background factors are shown as the upper integers for the individual 12 symptoms, in which attached “P” and “N” denote positive and negative association respectively. Standardized coefficients are shown as the lower decimal fractions. The levels of significance in these multivariate analyses were set at <0.05. The difference of associated background factors between H_2_RA users and digestive drug-free subjects were calculated; *p* scores below 0.05 were set for the level of significance.

To validate the differing results of the three groups (digestive drug-free subjects, PPI users, and H_2_RA users) with different population sizes, we calculated the effect sizes and power for all variables. The statistical power proved adequate in all analyses (Table S2). Most effect sizes are >0.02 with the exception of the four symptoms (g, i, k, and l) of digestive drug-free subjects group, but these four still display sizes of more than >0.015. The effect sizes of the explanatory variables for digestive drug-free users tend to be smaller than those for PPI users and H_2_RA users, but the difference is compensated by the smaller *p* value (*p*<0.005) for digestive drug-free users compared with gastric acid suppressant users (*p*<0.05).

### Our Five Proposed Categories of 12 Upper GI Symptoms Seem to Reflect the Differences of Background Factors between Digestive Drug-free Subjects and Gastric acid Suppressant Users

To accurately evaluate the differences between the digestive drug-free subjects and gastric acid suppressant users, statistical analysis (ANCOVA) was also performed.

For PPI users (*p* values in Table 3), two symptoms of the heartburn category (2 of 2) and two symptoms of the acid regurgitation category (2 of 3) have markedly different background factors compared to digestive drug-free subjects. One symptom of the pharyngo-upper esophageal discomfort category (1 of 2) has also significantly but not greatly different background factors. On the contrary, no symptoms of the dyspepsia category (0 of 4) and fullness while eating category (0 of 1) have significantly different background factors; in other words, associated background factors of the five symptoms in these two categories are quite similar between PPI users and digestive drug-free subjects.

For H_2_RA users (*p* values in Table 4), all five symptoms of the heartburn category (2 of 2) and acid regurgitation category (3 of 3) have significantly different background factors compared to digestive drug-free subjects. As shown in Table 3 and 4, *p* values of these five symptoms are quite similar between H_2_RA users and PPI users, with exception of “burping a lot (k)” of the acid regurgitation category. For the pharyngo-upper esophageal discomfort category, one symptom (1 of 2) has meaningfully but slightly different background factors, similar to PPI users. For the dyspepsia category, unlike PPI users, three symptoms (3 of 4) have significantly different background factors compared to digestive drug-free subjects.

Compared to the associated background factors of digestive drug-free subjects, those of PPI users and H_2_RA users are mostly similar, particularly for the symptoms of heartburn, acid regurgitation, and fullness while eating categories (Table 3 and 4). Conversely, associated background factors among gastric acid suppressant users are somewhat different for the symptoms of dyspepsia category (*p* values in Table 3 and 4). In this category, PPI users display similar associated factors to digestive drug-free subjects, whereas those of H_2_RA users are considerably different.

## Discussion

### Overview of Our Proposed Five Upper GI Symptom Categories and Significantly Associated Background Factors

For the heartburn category (f and a in Figure 3), strong associations with the two gastric mucosa-related factors (serum *HP* antibody (F4) and serum PG I/II ratio (F5) reflecting atrophic gastritis) observed in digestive drug-free subjects were for the most part not significant in gastric acid suppressant users (Table 2–4). Associations of the above-mentioned five common factors (F6–F10) in digestive drug-free subjects were also mostly not significant in gastric acid suppressant users, except for the strongest lifestyle-related factor “inadequate sleep” in H_2_RA users (F7 in Table 4). Associations of the three basic factors (age, gender, and BMI) for digestive drug-free subjects are similar to H_2_RA users and differ from PPI users (F1–F3 in Table 2–4). As a general rule for this category, associated background factors of digestive drug-free subjects and gastric acid suppressant users are completely different, which is clearly shown in the *p* values in Table 3 and 4.

For the dyspepsia category (b, c, e, and d in Figure 3), associations with “inadequate sleep (F7)” and the three basic factors (F1–F3) are strong in both digestive drug-free subjects and gastric acid suppressant users (Table 2–4). Marginal associations of the gastric mucosa-related factors (F3 and F4) in digestive drug-free subjects and gastric acid suppressant users are also similar to each other. As a general rule for this category, associated background factors of digestive drug-free subjects resemble to those of gastric acid suppressant users. Judging from the *p* values in Table 3 and 4, this resemblance is more evident for PPI users compared with H_2_RA users.

For the acid regurgitation category (l, j, and k in Figure 3), associations of the two gastric mucosa-related factors (F4–F5) and the five common factors (F6–F10) in Table 2 were mostly not significant in Table 3 and 4, except for “inadequate sleep (F7)” in H_2_RA users and “frequent skipping of breakfast (F10)” in PPI users. As in the heartburn category, associated background factors of digestive drug-free subjects are considerably different from those of gastric acid suppressant users. Judging from the *p* values in Table 3 and 4, this difference is more obvious in PPI users than H_2_RA users, except for the “burping a lot (k)” symptom.

For the pharyngo-upper esophageal discomfort and fullness while eating categories (g, i, and h in Figure 3), as in the dyspepsia category, a strong association with “inadequate sleep” is seen in both digestive drug-free subjects and gastric acid suppressant users (F7 in Table 2–4). In addition, positive and negative associations with the three basic factors of fullness while eating category are quite similar between digestive drug-free subjects and gastric acid suppressant users (F1–F3 in Table 2–4), which is also similar to the dyspepsia category to some extent.

### Differences of Associated Factors between Digestive Drug-free Subjects and Gastric Acid Suppressant Users may be Useful for Studying Appropriate Usages of PPI and H_2_RA upon Various Upper GI Symptoms

It is interesting that the background factors of symptoms belonging to the heartburn and acid regurgitation categories show statistically differences between digestive drug-free subjects and gastric acid suppressant users. On the contrary, background factors of symptoms belonging to the dyspepsia, pharyngo-upper esophageal discomfort, and fullness while eating categories show statistically no or small differences. In everyday clinical practice, the symptoms of heartburn and acid regurgitation tend to be well controlled with PPI or H_2_RA, whereas relief of the symptoms of other three categories tend to be difficult [36], [41]. The ease and difficulty to control each upper GI symptom seems to be related with similarity and difference of the significant background factors of each upper GI symptom between the digestive drug-free subjects and gastric acid suppressant users.

We are convinced that there should be some reason for these apparently different background factors of each upper GI symptom between the digestive drug-free subjects and gastric acid suppressant users. We hope that our finding will be a clue in elucidating the effects of attenuating gastric acid production or appropriate usage of PPI and/or H_2_RA against individual upper GI symptoms in the future.

### Study Limitations and Future Prospects

One limitation of our study is the cross-sectional design. We were therefore not able to perform accurate analyses of cause and effect. A second limitation of our study is insufficient data on the doses and types of gastric acid suppressants. More detailed information on orally taken PPIs and H_2_RAs might show more accurate relationships. A third limitation is possibility of unpredicted background factors which were not taken into account. Based on the many previous reports including ours [3], we selected the possibly important 13 factors, but we cannot deny the existence of other unknown factors strongly correlated with upper GI symptoms.

We plan to follow the present study cohort for at least ten years; the upcoming large-scale prospective analyses will help us confirm the true causative factors for individual upper GI symptoms. We believe that the practicability of our proposed categorization of upper GI symptoms will also be validated together with the time-course changes of upper GI symptoms and usages of PPI and/or H_2_RA. In addition, we are planning to evaluate the influence of *H. pylori* eradication [42] on our five upper GI symptom categories, because some symptom categories have significantly strong association with *H. pylori* infection status whereas others do not (Table 2). As many Japanese people with chronic *H. pylori* infection have undergone eradication therapy recently [5], [43], the effect of *H. pylori* eradication on various upper GI symptoms will become clear in our next report.

## Conclusions

The 12 typical upper gastrointestinal symptoms can be classified into heartburn, dyspepsia, acid regurgitation, pharyngo-upper esophageal discomfort, and fullness while eating symptom categories, which reflects various causative background factors. Differences between significantly associated factors of digestive drug-free subjects and digestive drug users may be useful for studying effects and usages of digestive drugs on various upper gastrointestinal symptoms.

## Supporting Information

Figure S1 **Distributions of 12 upper GI symptom scores in nine age groups and three BMI groups.** Respective upper GI symptom scores (from 0 to 4) are means of the data from 18,097 digestive drug-free subjects.
(TIF)

Figure S2 **Distribution of 12 upper GI symptom scores in regard of 11 background factors.** Respective upper GI scores (from 0 to 4) are means of the data from 18,097 digestive drug-free subjects.
(TIF)

Table S1 **Systemic univariate analyses of associations between the 12 upper GI symptoms and 13 background factors among the 18,097 digestive drug-free subjects.** The levels of significance in these univariate analyses were set at *p*<0.005. R means the Pearson’s correlation coefficient. Significant p-values are emphasized by shades of yellow.
(XLS)

Table S2 **The effect sizes and power for the 13 variables in the three multivariate analyses concerning the 18,097 digestive drug-free users, 364 PPI users, and 528 H_2_RA users.**
(XLS)
