# Supplementary figures and images for: Categorization of Upper Gastrointestinal Symptoms Is Useful in Predicting Background Factors and Studying Effects and Usages of Digestive Drugs
Source: PLoS One. 2014 Feb 5;9(2):e88277. doi: 10.1371/journal.pone.0088277 (PMC3914954; doi:10.1371/journal.pone.0088277)

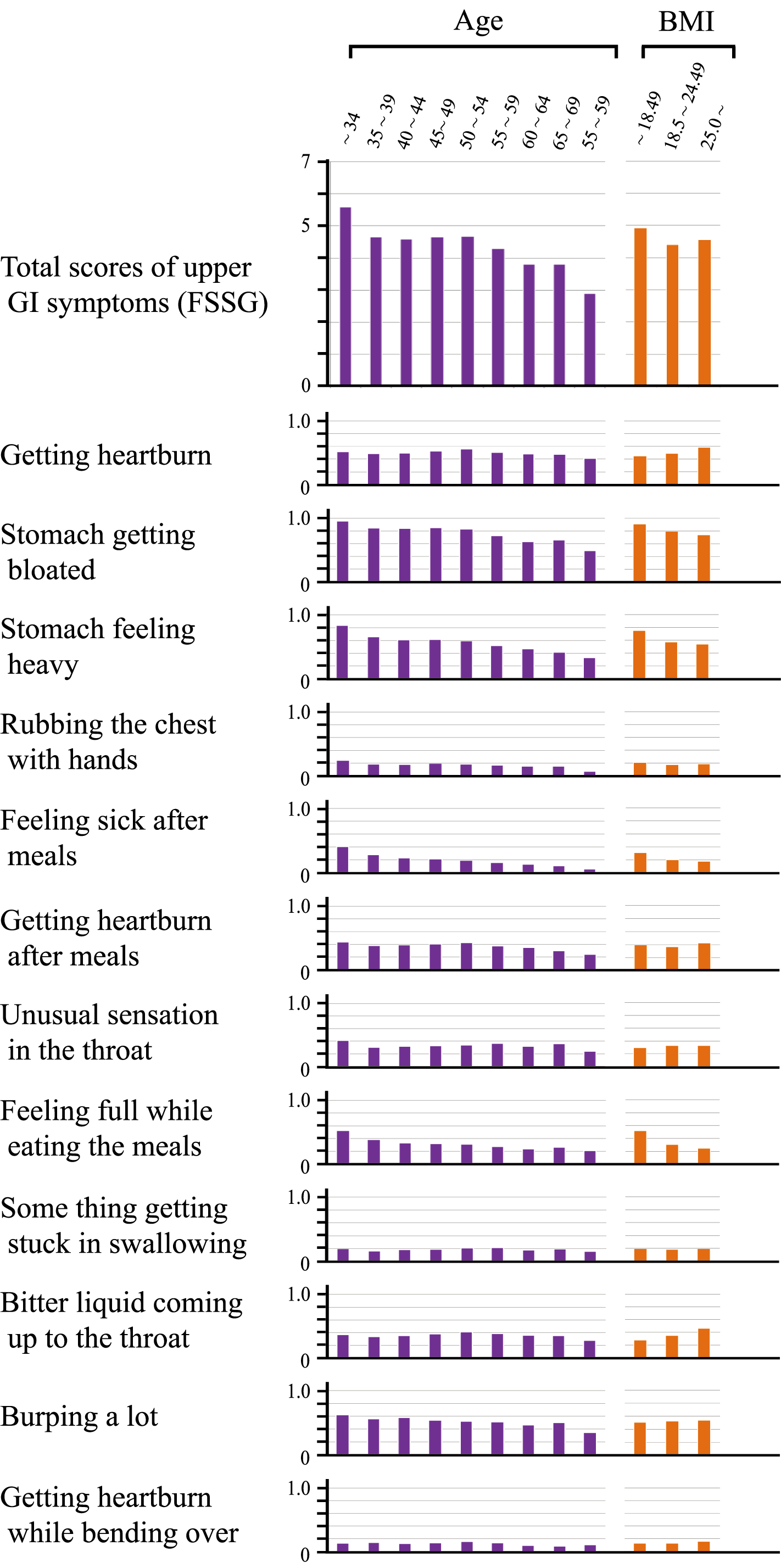

Supplement: Figure S1 — Distributions of 12 upper GI symptom scores in nine age groups and three BMI groups. Respective upper GI symptom scores (from 0 to 4) are means of the data from 18,097 digestive drug-free subjects. (TIF) [file pone.0088277.s001.tif]

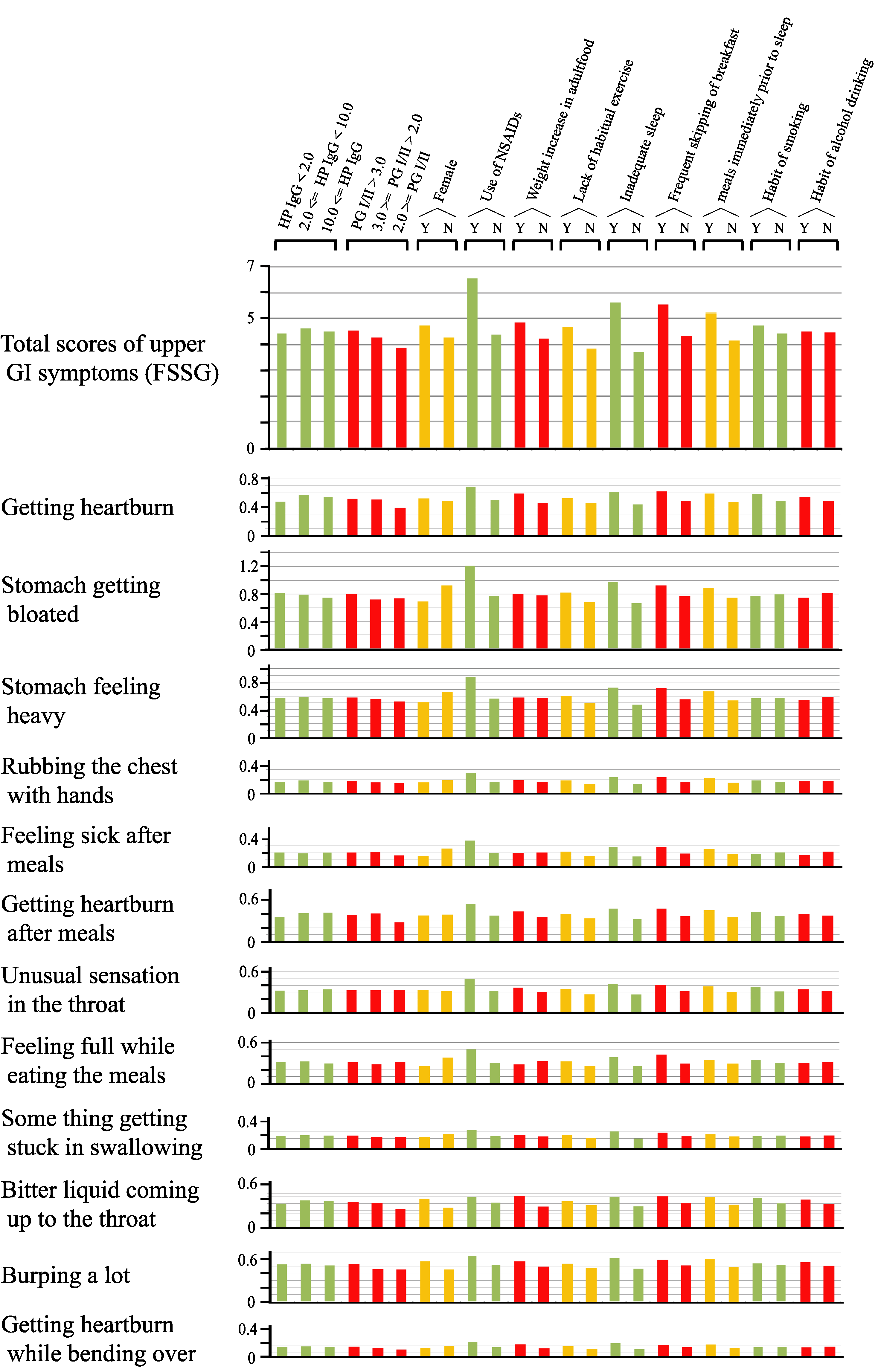

Supplement: Figure S2 — Distribution of 12 upper GI symptom scores in regard of 11 background factors. Respective upper GI scores (from 0 to 4) are means of the data from 18,097 digestive drug-free subjects. (TIF) [file pone.0088277.s002.tif]
